# Safety and efficacy study of allogeneic human menstrual blood stromal cells secretome to treat severe COVID-19 patients: clinical trial phase I & II

**DOI:** 10.1186/s13287-022-02771-w

**Published:** 2022-03-07

**Authors:** Mina Fathi-Kazerooni, Samrand Fattah-Ghazi, Maryam Darzi, Jalil Makarem, Reza Nasiri, Faeze Salahshour, Seyed Ali Dehghan-Manshadi, Somaieh Kazemnejad

**Affiliations:** 1 Nanobiotechnology Research Center, Avicenna Research Institute, ACECR, Tehran, Iran; 2 Department of Anesthesiology and Intensive Care, Imam Khomeini Hospital Complex, Tehran University of Medical Sciences, Tehran, Iran; 3 Avicenna Fertility Clinic, Avicenna Research Institute, ACECR, Tehran, Iran; 4 Department of Radiology, Advanced Diagnostic and Interventional Radiology Research Center (ADIR), Tehran University of Medical Sciences, Tehran, Iran; 5 Liver Transplantation Research Center, Imam Khomeini Hospital Complex, Tehran University of Medical Sciences, Tehran, Iran; 6 Department of Infectious Diseases and Tropical Medicine, Imam Khomeini Hospital Complex, Tehran University of Medical Sciences, Tehran, Iran

**Keywords:** COVID-19, Menstrual blood stromal cells, Secretome, GMP, New treatment

## Abstract

**Background:**

Cell-free Mesenchymal stromal cells (MSCs) have been considered due to their capacity to modulate the immune system and suppress cytokine storms caused by SARS-CoV-2. This prospective randomized double-blind placebo-controlled clinical trial aimed to assess the safety and efficacy of secretome derived from allogeneic menstrual blood stromal cells (MenSCs) as a treatment in patients with severe COVID-19**.**

**Methods:**

Patients with severe COVID-19 were randomized (1:1) to either MenSC-derived secretome treatment or the control group. Subjects received five intravenous infusions of 5 mL secretome or the same volume of placebo for five days and were monitored for safety and efficacy for 28 days after treatment. Adverse events, laboratory parameters, duration of hospitalization, clinical symptom improvement, dynamic of O_2_ saturation, lymphocyte number, and serial chest imaging were analyzed.

**Results:**

All safety endpoints were observed without adverse events after 72 h of secretome injection. Within 28 days after enrollment, 7 patients (50%) were intubated in the treated group versus 12 patients (80%) in the control group. Overall, 64% of patients had improved oxygen levels within 5 days of starting treatment (*P* < 0.0001) and there was a survival rate of 57% in the treatment group compared to 28% in the control group was (*P* < 0.0001). Laboratory values revealed that significant acute phase reactants declined, with mean C-reactive protein, ferritin, and D-dimer reduction of 77% (*P* < 0.001), 43% (*P* < 0.001), and 42% (*P* < 0.05), respectively. Significant improvement in lymphopenia was associated with an increase in mean CD4^+^ and CD8^+^ lymphocyte counts of 20% (*P* = 0.06) and 15% (*P* < 0.05), respectively. Following treatment, percentage of pulmonary involvement showed a significant improvement in the secretome group (*P* < 0.0001). This improvement differed significantly between survivors and those who were dying (*P* < 0.005).

**Conclusions:**

For the first time, this study demonstrated that in hospitalized patients with severe COVID-19, therapy with MenSCs-derived secretome leads to reversal of hypoxia, immune reconstitution, and downregulation of cytokine storm, with no adverse effects attributable to the treatment. Given these outcomes, it may be possible to use this type of treatment for serious inflammatory lung disease with a mechanism similar to COVID-19 in the future. However, it is necessary to evaluate the safety and efficacy of MenSCs-derived secretome therapy in clinical trials on a larger population of patients.

*Trial registration*: ClinicalTrials.gov Identifier: NCT05019287. Registered 24AGUEST 2021, retrospectively registered, https://clinicaltrials.gov/ct2/show/record/NCT05019287. IRCT, IRCT20180619040147N6. Registered 04/01/2021.

## Introduction

In late 2019, a new viral illness caused by the severe acute respiratory syndrome coronavirus 2 (SARS-CoV-2) began in China and quickly became a global pandemic [1]. To date, efforts have been made to treat acute respiratory distress syndrome (ARDS) caused by Coronavirus disease 2019 (COVID-19) with non-invasive supplemental O_2_ and delay the intubation as long as possible. Several studies have demonstrated that in this group of patients, early therapeutic intervention may reduce the risk of developing the disease into hypoxia, which requires intubation and mechanical ventilation and is associated with a mortality rate of approximately 67–94% [2–4]. Available evidence suggests that uncontrolled excessive production of soluble inflammatory markers induces a “cytokine storm” playing a key role in the development of COVID-19ARDS [5]. In both types of classic ARDS and COVID-19ARDS, there are noticeable levels of pro-inflammatory biomarkers, increased capillary endothelial permeability, and a rise in inflammatory cells counts in the vascular and alveolar compartments [6]. Nevertheless, there are significant differences in the type of enhanced markers, so that less expression of IFNs and increased thrombotic mediators occurred in COVID-19 ARDS compared to classical ARDS [6, 7]. High levels of pro-inflammatory cytokines such as interleukin (IL)-1β, IL-2, IL-6, IL-7, IL-8, IL-17, and tumor necrosis factor-α (TNF-α) have been reported in the serum of patients with severe COVID-19, which is associated with mortality risk [8]. Many scientists and researchers are now attempting to identify the most effective drug or combination against the disease in the context of randomized clinical trials. At present, several agents such as antivirals (including remdesivir), hydroxychloroquine, monoclonal antibodies, antisense RNA, corticosteroids, anticoagulants, and convalescent plasma are being assessed [9, 10]; however, the efficacy of some of them, such as chloroquine or its hydroxyl analogue in treating this disease, is debatable in various studies [11]. In addition to these drug therapies, the use of MSC treatment because of its properties as adjunctive therapy has been considered by researchers in preclinical studies of lung diseases [12, 13]. In several animal models of ARDS, demonstrated that paracrine release of different soluble products by MSC could induce anti-inflammatory, immunomodulatory, and anti-apoptotic effects, improve epithelial and endothelial cell recovery, enhance microbial and alveolar fluid clearance, and prevent tissue fibrosis thus resulting in improved lung and distal organ function and survival [14–17]. Furthermore, this type of cell therapy has been safe and effective in early-stage clinical trials. Despite limitations such as small sample sizes, heterogeneity of source, dose, route, and frequency of MSC administration, these clinical studies showed that MSC administration was safe and well tolerated and, in most cases, resulted in an improved hemodynamic and respiratory state associated with a reduction in pulmonary and systemic inflammatory biomarkers [18–22]. On the other hand, derivatives of these cells, including cell secretome with unique properties, have been evaluated in some studies [23–25]. The secretome of MSC is a complex mixture of soluble protein products consisting of growth factors, cytokines, microvesicles, and exosomes [26]. Trophic factors in the MSC secretome include tumor growth factor (TGF)-β, hepatocyte growth factor (HGF), vascular endothelial growth factor (VEGF), leukemia inhibitory factor (LIF), epidermal growth factor (EGF), nerve growth factor (NGF), Prostaglandin (PG) E2, interleukin-1 receptor antagonist (IL-1Ra), Metalloproteinase-processed-C motif chemokine ligand2 (mpCCL2), and brain-derived neurotrophic factor (BDNF) [27–29]. Because the soluble factors secreted by the MSCs appear to repair tissue damage, reduce inflammation, and increase tissue repair capacity, the current vision using cell-free strategies such as that proposed by the MSC secretome offers key benefits on cell transplantation [11]. The major advantage of using cell-free strategies is due to the ability of these products to exert similar effects of those from parental cells without a potential risk of immunogenicity (graft versus host disease [GvHD], for instance). Another important advantage of cell-free strategies is that the cells may form clusters and lead to thromboembolic events, which are especially important in dealing with people with severe COVID-19. Other benefits of using MSC-based cell-free derivatives include no worries about cell survival after transplantation, lower levels of cell surface protein expression, less immunity, and no need for large numbers of cells for injection (1 × 10^6^ cells/kg/dose) and ultimately easier, more convenient and more cost-effective mass production with the possibility of monitoring the safety, effectiveness and dosage of the final product like conventional drugs [30]. Several preclinical studies have shown that MSC-secreted extracellular vesicles (EVs) function similarly or even more effectively than mesenchymal stem cells themselves in controlling of lung injury caused by inflammation [31, 32]. These EVs are about 30–170 nM in size that express CD81, CD63, and tumor susceptibility gene (TSG) 101 [33, 34].

Based on current knowledge about the therapeutic properties of MSC derivatives on inflammatory lung disease, an increasing number of experimental studies have examined the effectiveness of MSC-based cell-free products in COVID-19 patients in order to find an effective treatment to control severe inflammation in patients [35]. According to the data, we hypothesized MSC-derived secretome can be a new next-generation, multitarget biological agent that could be the key to cytokine storm regulation and the reversal of host antiviral defenses associated with COVID-19.

Among the different types of MSCs, menstrual blood-derived stromal cells (MenSCs) have been considered in numerous pre-clinical and clinical studies including treatment of pulmonary diseases in recent decades because of their unique characteristics, such as high proliferation rate, low immunogenicity, and non-invasive periodical collection [36–39]. Earlier studies conducted on the secretome content of MenSCs by this group within parallel with other groups demonstrated that these cells secrete plenty of bioactive molecules, especially anti-inflammatory interleukins and cytokines, growth factors and EVs [39, 40] which proposes the potential effect of MenSCs derived secretome in treatment of various diseases and disorders including inflammatory and pulmonary diseases [41]. Therefore, this study focused on the compatibility of these cells in the treatment of COVID-19 patients. MenSC-derived secretion was tested for sterility and prepared in facilities that comply with current Good Manufacturing Practices (cGMP) and then was administered to 14 patients with severe Covid-19 pneumonia. After injecting five doses of MenSC-derived secretion intravenously, the safety of this type of treatment in patients was assessed by evaluating perfusion reactions and any side effects. The efficacy of secretome injection was also evaluated by assessment of overall condition, as demonstrated by blood oxygen saturation and oxygen requirements, degree of inflammation, and immunocompetence, indicated by levels of C-reactive protein (CRP), lactate dehydrogenase (LDH), D-dimer, ferritin, and cell counts of the cluster of differentiation (CD)4^+^ and CD8^+^ T lymphocytes.

## Materials and methods

### Trial design

The patients were enrolled in Phase I and II randomized controlled clinical trial for evaluation of the safety and efficacy of infusions of secretome derived from the MenSCs. All patients with severe pneumonia due to COVID-19 were admitted to the intensive care unit (ICU) at the Imam Khomeini Hospital Complex from April 17 to June 7, 2021.

Written informed consent was obtained following the initial consultation with the patient. All the procedures in this clinical trial study were conducted according to Good Clinical Practice (GCP) or GMP guidelines approved by the Biomedical Research Ethics Committee of Academic Center for Education, Culture, and Research (ACECR) (Code:IR.ACECR.REC.1399.005). The study was registered at the Iranian Registry of Clinical Trials (IRCT20180619040147N6) and the cell manufacturing was performed by Zayabiotech Company (Tehran, Iran) in GMP cleanroom authorized by Iran food and drug administration authorities.

### Participants

Subjects diagnosed with COVID-19 ARDS were eligible for inclusion if they met the eligibility criteria. The inclusion criteria were age 25–75 years, positive result on SARS-CoV-2 polymerase chain reaction (PCR), RR > 30 times/min, resting oxygen saturation of 90% or less, arterial partial pressure of oxygen/oxygen concentration ≤ 300 mmHg, and pulmonary infiltration greater than 50% in 24–48 h. Exclusion criteria were pregnancy or breastfeeding, history of drug reactions, pneumonia caused by bacteria, Mycoplasma, Chlamydia, Legionella, fungi or other viruses, airway obstruction due to lung cancer or unknown factors, Carcinoid syndrome, long-term use of immunosuppressive drugs, hemodialysis or peritoneal dialysis, creatinine clearance < 15 mL/min, moderate to severe liver disease (Child–Pugh score > 12), deep vein thrombosis (DVT) or pulmonary embolism over the past 3 years, being under high-frequency oscillatory ventilation support (ECMO),and patients with HIV, hepatitis B virus, or hepatitis C virus infections. All patients were already initiated on remdesivir, corticosteroid, and anticoagulants.

### Randomization

**Fig. 1:**
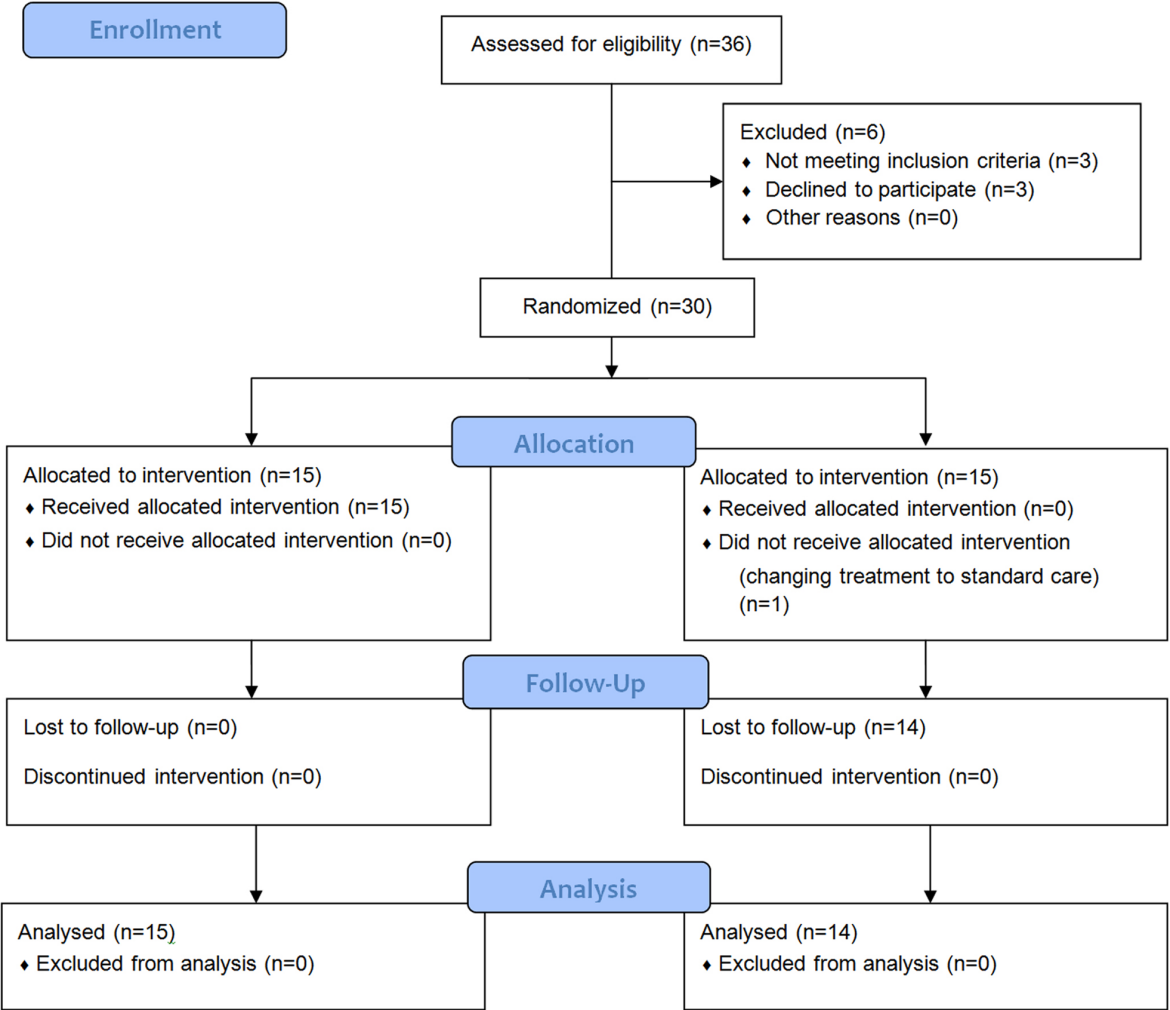
Flow diagram showing enrollment, allocation, follow up and analysis of patients

A total of 36 patients were considered for eligibility; 30 patients who met the acceptance criteria were randomized 1:1 to receive either MenSCs-secretome (*n* = 15) or the control (injectable normal saline) (*n* = 15) (Fig. 1).In this study, a simple computer-assisted randomization method was used, in which a computer-generated list of numbers from 1 to 30 was prepared. Depending on the time of hospitalization, one of these numbers has been allocated to the patient, and based on the created list; patients were assigned to the treatment and control groups.

### Blinding

The study was double-blinded: the patients and the data analysts were blinded to group assignment.

### MenSC-derived secretome investigational product

MenSCs were manufactured as previously described [37]. MenSCs were culture-expanded from a previously established and characterized master cell bank (MCB) derived from the menstrual blood collected from at least 5 healthy women [37]. The quality of the MenSCs stored in the bank was tested according to the applicable U.S. Food and Drug Administration (FDA) regulations for fungal and bacterial contamination using sterility by direct inoculation, mycoplasma assay by both culture and PCR (complaint with USP chapter 〈63〉 Mycoplasma Tests), endotoxin test (according to USP chapter 〈85〉 Bacterial Endotoxins Test), karyotyping with routine G-banded chromosomes analysis, and immunophenotyping assessment through expression analysis of CD73, CD44, CD90, and CD45.

In preparation for infusion, frozen MenSCs were quickly thawed and cultured up to 70% confluence in Dulbecco's Modified Eagle Medium/Nutrient Mixture F-12 (DMEM-F12) containing platelet lysate (PL). The medium was discarded and, following three washes with phosphate-buffered saline (PBS), the cells were incubated with red phenol-free DMEM-F12 at 5% CO_2_ at 37 °C for 48 to 72 h. After that, the supernatant was aspirated, pooled, and sterile filtered through a 0.2-μm syringe filter (Membrane Solutions) and centrifuged at 2000 rpm for 5 min at RT. Finally, the supernatant was aspirated (soluble fraction) and packed in 5 mL microtubes.

### Interventions

Subjects in the MenSC-derived secretome treatment group (*n* = 14) received five intravenous infusions of 5 mL of MenSCs-derived secretome diluted in 100 mL of normal saline for 5 consecutive days for 60 min. The control group received five infusions of 100 mL of normal saline for 5 consecutive days. The best standard of care was provided in both groups, consistent with current institutional COVID-19 guidelines.

### Assessments

Before starting the first infusion, the basic parameters including the following experiments were measured: CBC, PT/INR, LFT, ESR, CRP, ferritin, D-dimer, T lymphocyte panel, chest Computed tomography (CT)-scan, and ECG. During the injections and 1 h later, vital signs were recorded under the ICU standard protocol. Laboratory collection and clinical assessment were performed before each infusion and repeated daily up to 28 days following treatment or until the last day of hospitalization. The primary endpoint of the trial was safety: adverse events within six hours; cardiac arrest or death within 24 h of every infusion. Secondary endpoints included patient survival at 28 days after initial infusion and time to recovery.

Peripheral blood CD4 and CD8 markers were tested using flow cytometry procedure. Briefly, 100 µL of whole blood was poured into three separate test tubes, each containing 10 µL of Anti-Hu CD4 PE (Exbio, Czech Republic), CD8 FITC (Exbio, Czech Republic), and Anti-Hu antibodies. CD45 PerCP (Cytognos, Spain) was mixed well and incubated at room temperature for 30 min. The red blood cells were lysed using RBC lysis buffer solution (APRAD, Iran) for 5 min at 300 g. The supernatant was then discarded, and the cells were suspended with 0.3–0.5 mL PBS. The samples were immediately read using flow cytometry (SysmexPartec Pas III, Germany).

For evaluation of pulmonary involvement, chest CT scan images were obtained at the time of admission and discharge, lying on the back and fully inspired without contrast. Chest CT scan findings were recorded according to the Fleischner Society glossary and published literature on viral pneumonia [42]. Chest CT scan features included (a) predominant pattern: ground-glass opacification/opacity (GGO), consolidation, or mixed; (b) dominant distribution pattern: peripheral (peripheral one-third of the lung)/pleural based, peripheral/pleural sparing, axial (medial two-thirds of the lung), or diffuse; (c) the number of involved lobes; (d) other morphologies: crazy paving, reverse halo sign, intralesional traction bronchiectasis, parenchymal band, and Mesh-like opacity; and (e) additional findings: underlying pulmonary diseases such as bronchiectasis, emphysema, interstitial lung disease, cardiomegaly, pleural effusion (unilateral or bilateral), subsegmental atelectasis, mediastinal or hilar lymphadenopathy, pericardial effusion, and pleural thickening.

### Statistical analysis

The study population consisted of all patients who had received all their treatment doses and whose clinical information was available for at least 10 days after starting treatment. The frequency tables were used to describe the individual data. Before and after treatment datasets were evaluated in each group using a paired *t* test analysis in GraphPad PRISM 6.0. Analysis of one variable at several intervals was performed by repeated-measures ANOVA test. Comparison between control and intervention groups was performed using an unpaired *t* test. The Kaplan-Meyer curve was used for survival analysis.

## Results

### Baseline patient characteristics

Table 1 summarizes basic demographic and clinical characteristics for patients. 9 men and 6 women in the intervention group and 10 men and 5 women in the control group were enrolled in the study. One patient in the intervention group discontinued the study after receiving a dose of the secretome. In the intervention group, type 2 diabetic patients comprised 21.5% of the population whereas hypertension comprised 28.5%. In contrast, in the control group, 26.6% of patients had diabetes type 2 and 33.3% of patients had hypertension.

**Table 1:** Baseline demographics and pretreatment conditions of enrolled participants

Baseline demographics and pretreatment conditions of enrolled participants			
		Treated group	Control group
Age (years)		46.43 ± 11.91	53.67 ± 10.30
Gender	Male	9	10
	Female	5	5
O_2_ support category	Mechanical ventilation	2	2
	NIV	5	6
	Reserve mask	7	7
Illness before admission	Duration (days)	12.14 ± 7.6	10.27 ± 4.9
Illness before treatment	Duration (days)	16.0 ± 7.8	12.67 ± 5.5
Pre-existing comorbidities	T2DM	3	4
	HTN	4	5
	Any condition	2	1
SpO_2_	85–90	2	6
	80–84	3	3
	˂ 80	9	6
CT involvement percent	50–75%	7	9
	˃ 75%	7	6

### Safety

No allergic reactions to the injection or side effects were observed within the first five days of the treatment. Furthermore, no adverse effects attributed to the effects of secretome infusion were reported during the 28-day follow-up of patients. Side effects over the 28-day follow-up period include aggravating hypoxic respiratory failure requiring intubation (*n* = 7), pulmonary embolism (*n* = 3), myocardial infarction (*n* = 1), sepsis (*n* = 1), and death (*n* = 6) that all these deaths occurred in intubated patients. After assessing the cause of death and the time interval of deaths from the time of injection, all events were logically related to the progression of COVID-19 disease and were not directly related to this type of treatment.

### General clinical implications

**Fig. 2:**
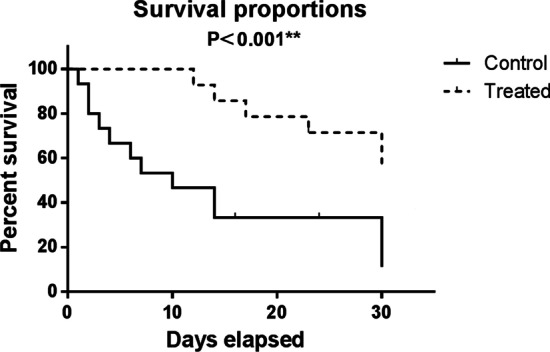
Kaplan–Meier survival curves for overall survival in control and treated groups which showed that the difference in survival rate between these two groups was statistically significant (*P* < 0.001)

The survival rate in this study was 57% in the intervention group and 20% in the control group, which differed significantly (*P* < 0.001) as shown in the Kaplan Meyer diagram (Fig. 2). In total, all non-intubated patients, who constituted 50% of the patients, all improved (7/14) and were discharged from the hospital after an average of 12.3 ± 3.68 days after the first dose infusion. In total, 50% of patients with higher disease severity or underlying diseases, especially diabetes type 2, needed intubation and mechanical ventilation, one of whom was extubated and released and six died. By comparison, in the control group, out of 15 patients, only 3 patients recovered and were discharged from the hospital after a mean of 17.0 ± 4.35 days after the start of the intervention.

### Oxygenation

Oxygenation was assessed by calculating the percentage of SpO_2_ at room temperature, and then with oxygen support as well as tracking the oxygen demand first, on the days of the infusion and 28 days after the infusion of the first dose. Overall, 64% of patients had improved oxygen levels within 5 days of starting treatment. The average increase in the percentage of oxygenation from the beginning to the 14th day after the start of treatment or the last day of hospitalization was 21.3% (*P* < 0.0001).

### Laboratory data

**Fig. 3:**
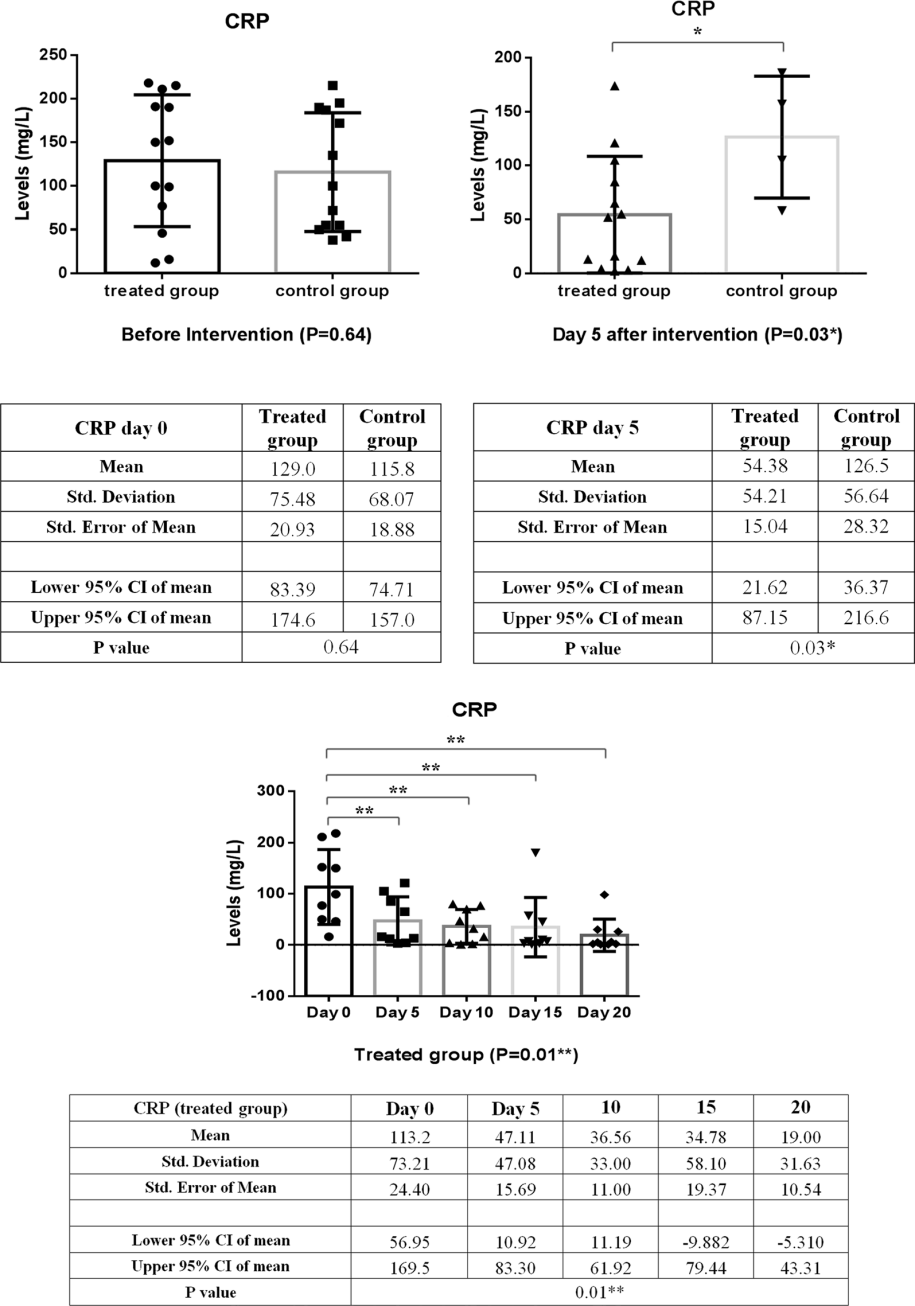
Change in acute phase reactant (CRP) levels before and after of secretome administration. Mean reduction of CRP in treated group was 77% (*P* < 0.001)

**Fig. 4:**
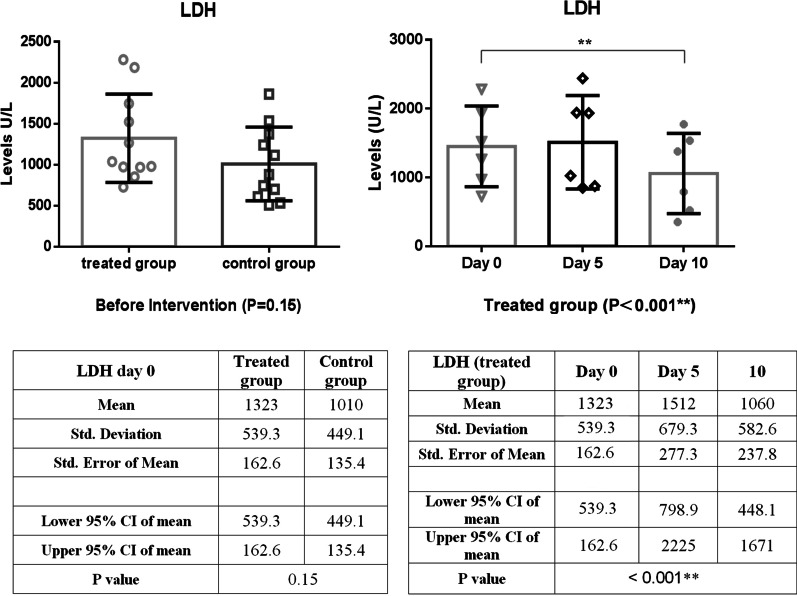
Decreasing trend of LDH level in the treated group within 10 days of starting treatment. A significant decrease in LDH levels was observed 10 days post-treatment (*P* < 0.001)

**Fig. 5:**
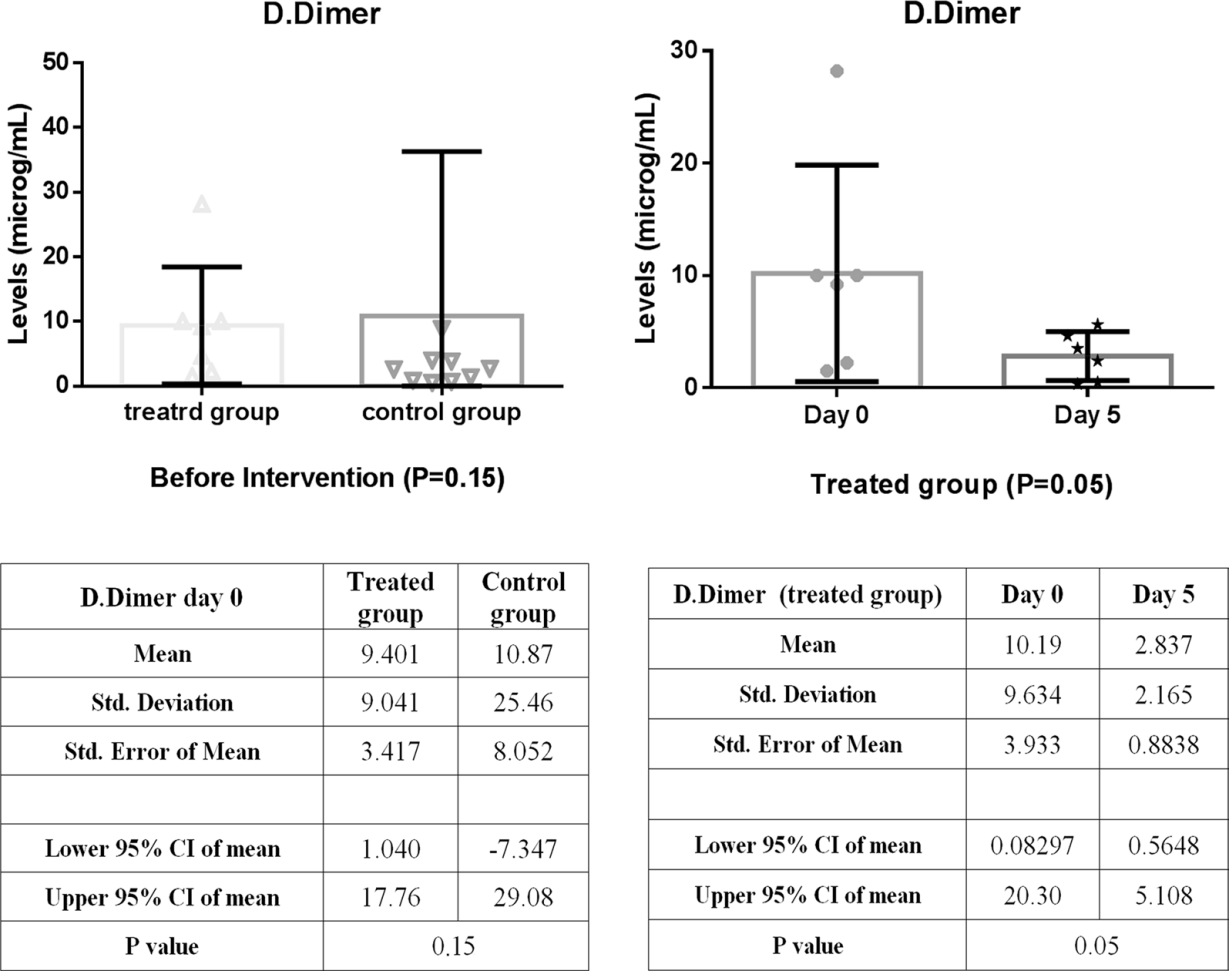
Decreasing trend of D.Dimer level in the treated group within 5 days of starting treatment. Mean reduction of D-dimer in treated group was 42% (*P* < 0.05)

**Fig. 6:**
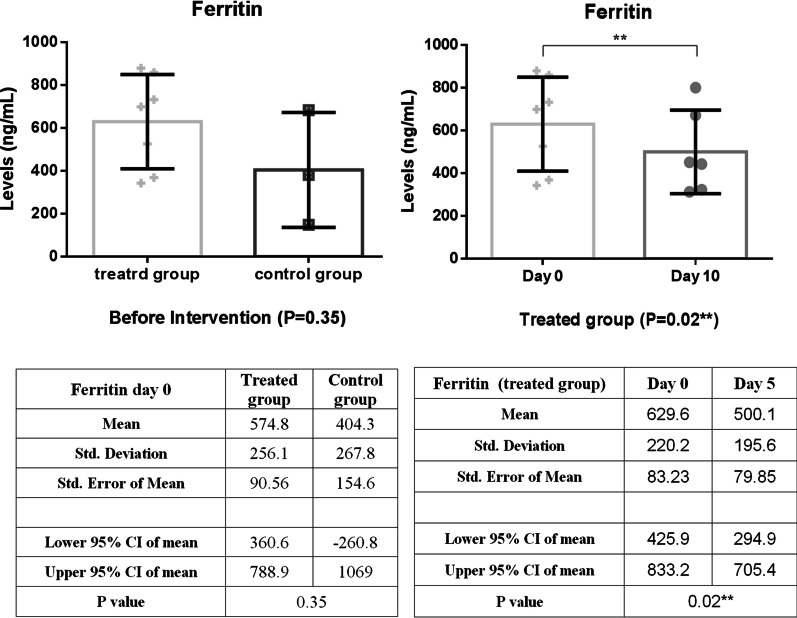
Decreased trend of ferritin level in the treated group within 5 days of starting treatment. Mean reduction of ferritin in treated group was 43% (*P* < 0.001)

**Fig. 7:**
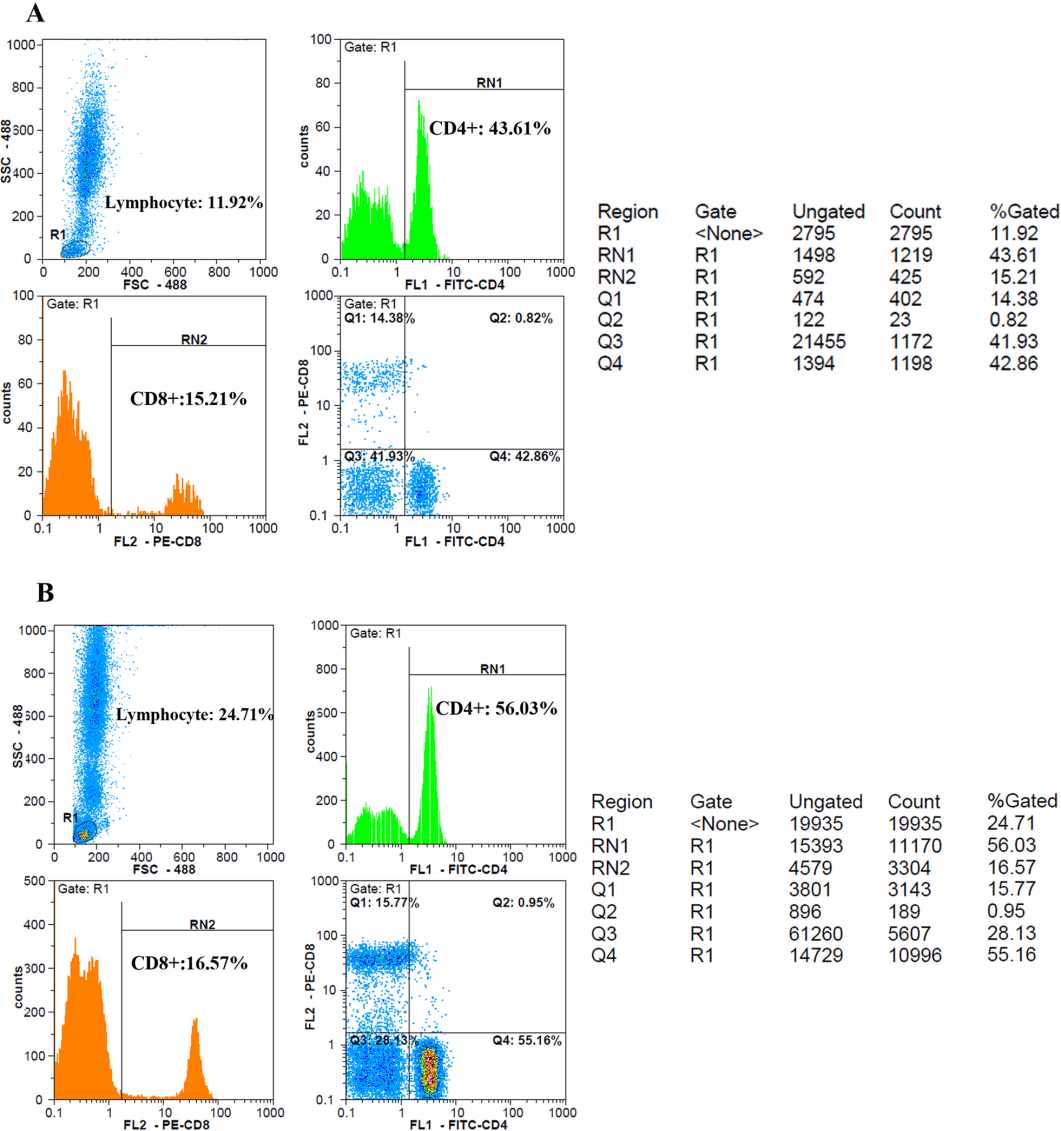
Flow cytometry gating strategy for CD4^+^ and CD8^+^ cells. Lymphocytes were first identified by R1 gate in forward scatter (FSC) and side scatter (SSC) graph. CD4^+^ and CD8^+^ T cells were then measured by means of anti CD4 and anti CD8 antibodies, labeled by FITC and PE respectively; as illustrated in the histogram graphs separately. **A** and **B** represent the changes in CD4^+^ and CD8^+^ lymphocyte counts between 5 and 10 days after secretome administration in a 35-year-old male patient, respectively

In comparison, between intervention and control groups, there was a significant reduction in CRP levels over the first five days of treatment, as shown in Fig. 3 (*P* = 0.03). In addition, the CRP level of the intervention group decreased significantly (*P* = 0.01) during the follow-up period with small fluctuations (Fig. 3). As shown in Fig. 4, LDH concentration decreased more slowly than those of CRP in the treated group; as a result, a statistically significant decrease in LDH level was observed 10 days post-treatment (*P* < 0.001). A similar reduction in D. Dimer level was observed in the intervention group within 5 days of treatment (Fig. 5). The decrease in serum ferritin concentration was the lowest among the serum markers in the treated group; a notable decrease in ferritin concentration occurred after 10 days, as shown in Fig. 6 (*P* = 0.02). There was an increase in the number of lymphocytes including subsets staining positive for CD4^+^ and CD8^+^ on flow cytometry compared to baseline values with 10 days post secretome treatment, which is a statistically significant increase in the number of CD8^+^ lymphocytes (*P* = 0.03, Fig. 7).

### CT scan

At the time of recruitment, a total of 28 CT scans [14 for the treated group and 14 for the control group] were performed. The most common finding was GGOs, appearing in 85.8% of the treated group and 66.7% of the control group (*P* = 0.55), followed by consolidation in 2 patients (25.7%) in the first group and 1 patient (34.6%) in the second group (*P* = 0.33). The dominant diffuse distribution was noted in 85.8% of patients of the treated group and 83.3% of patients of the control group (*P* = 0.89). The crazy paving sign was observed in 78.6% of patients of the intervention group and 86.7% of patients of the control group (*P* = 0.56). Parenchymal bands (57.1% vs. 40.0%, *P* = 0.36), bronchiectasis (12.3% vs. 20.0%, *P* = 0.74), subsegmental atelectasis (28.6% vs. 46.6%, *P* = 0.51), target sign (14.3 vs. 33.3, *P* = 0.23), and air bronchogram (14.3% vs. 40.0%, *P* = 0.12) were reported in treated and control group, respectively. Other findings include mosaic pattern, pulmonary emphysema, centrilobular nodules, bronchogenic carcinoma, peribronchial thickening, interlobular septal thickening, cavity, mediastinal lymphadenopathy, halo sign, alveolar collapse, pulmonary embolism, and infarction were seen neither in the treated nor in the control group.

**Fig. 8:**
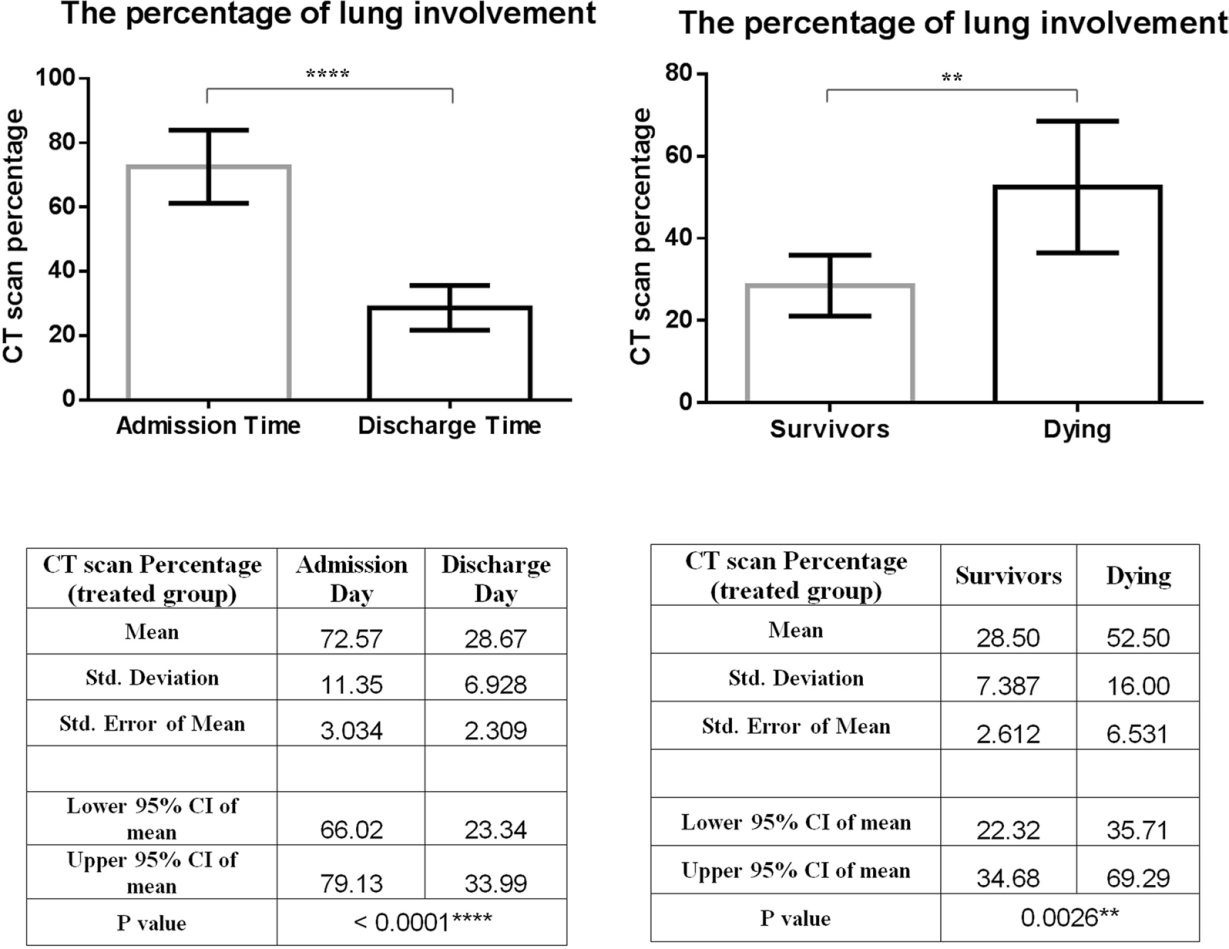
Change in the CT scan percentage of lung involvement before and after of secretome administration. This treatment significantly improved the lung involvement of patients when they were discharged from hospital. Furthermore, this effect was completely different in both groups of surviving and dying patients

**Fig. 9:**
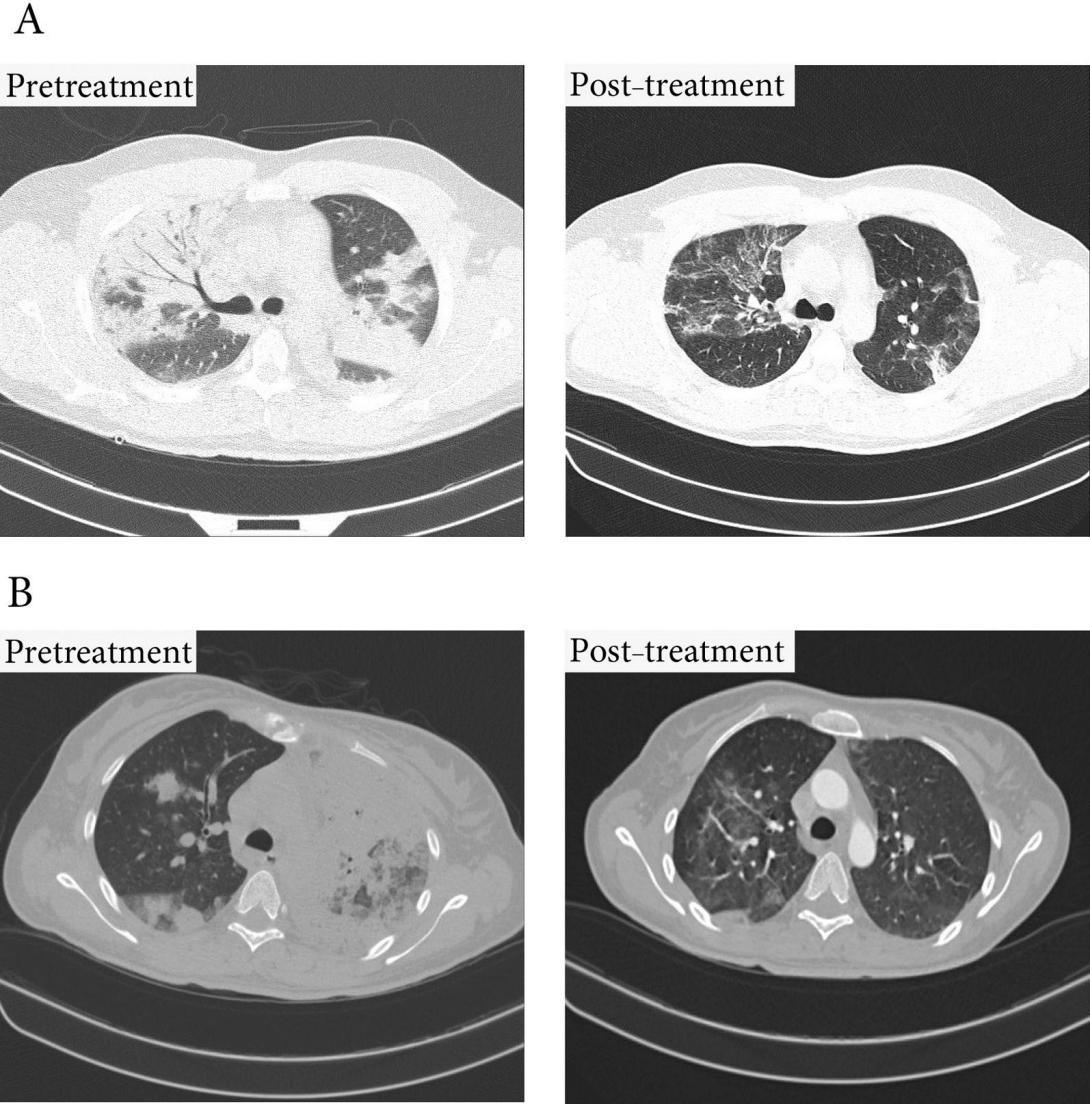
Lung CT scan. **A** Significant improvement in lung lesions within 10 days of starting treatment in a 37-year-old male patient **B** Significant improvement in lung lesions within 5 days of starting treatment in a 45-year-old female patient

At the outset of the study, there was no significant difference in the percentage of pulmonary involvement between the intervention and control groups (72.6 ± 11.3 vs. 66.8 ± 9.9, *P* = 0.33). However, following treatment, this rate showed a significant improvement in the MenSCs-derived secretome group (72.6 ± 11.3 vs. 28.7 ± 6.9, *P* < 0.0001) (Fig. 8). This improvement varied considerably between survivors and those dying (28.7 ± 6.9 vs. 46.7 ± 11.7, *P* = 0.002) (Fig. 9). Chest CT images of the two COVID-19 patients are demonstrated in Fig. 8.

## Discussion

The results of this prospective, double-blind clinical trial in patients with severe COVID-19 have demonstrated that the secretome of menstrual blood stromal cells can be safely administered by intravenous injection. In none of the patients, the injection of this cell-free product resulted in infusion reactions. We received no reports of immediate (< 6 h), intermediate (< 24 h), or delayed (< 72 h) adverse reactions. A review of adverse events after this period revealed that there was no association between the occurrence of these accidents and the therapeutic intervention of this product.

Reports of ICU mortality due to COVID-19 around the world showed that more than 70% of the patients with criteria of severe pneumonia will be intubated and need mechanical ventilation support [43, 44], which will be associated with 50–97% mortality [1]. On the other hand, the risk of mortality in patients requiring noninvasive oxygen support is estimated as high as 60–79% [3, 45]. Based on this data, along with the condition of our patients, we expected that most patients would require intubation within 24 to 48 h if treatment was not initiated. But evidence has shown that with the onset of treatment, only half of the 14 patients reached this stage. It can therefore be concluded that the use of the secretome can play a preventative role in the progression of the disease toward the need for invasive oxygen support and mechanical ventilation, although further studies at subsequent stages of this randomized controlled trial are necessary to prove efficacy. A total of 57% of treated patients (8/14) recovered, suggesting a profound reversal of disease progression and indicating the optimal time for administration of the MenSCs secretome is the early phase of the cytokines storm. Overall, treatment with this cell-free product has been linked to a significant 29% improvement in patient oxygenation (*P* < 0.0001) and demonstrated a reduced need for oxygen support within 48–72 h of commencing treatment.

It should be noted that even in patients who eventually died in this study, several clinical parameters, including oxygenation and inflammatory markers, showed the optimal initial response to the injection, indicating the greatest reduction in the severity of inflammation following the third injection. This may suggest that booster doses may be necessary for these patients to achieve the desired anti-inflammatory response. Perhaps the short half-life of the secretome components [30] and the over-activity of the inflammatory system in these critically ill patients may be a justification for the need to infuse higher doses of this drug. However, in preclinical studies of exosome-based therapies, Evidence has been obtained of the alignment of the effect of circulating proteases on the inactivation of exosomal products at intervals from the time of injection [46].

In this study, changes such as a significant increase in the number of CD4^+^ and CD8^+^ T cells were observed, which led to a decrease in lymphopenia in patients, as well as a significant reduction in all acute inflammatory factors of serum and optimal improvement of pulmonary inflammation in patients. Infiltration of radiological images showed that, as expected, modulation of the hyperactive immune system is the most important mechanism of action of this cell-free treatment.

Exact mechanisms underlying efficiency of MenSCs derived secretome are not clear. However, it could be attributed to anti-inflammatory, anti-apoptotic and anti-fibrotic effects of the cytokines, interleukins, growth factors and EVs in the administrated secretome. In our study, to determine the underlying mechanisms of the proliferation of natural killer cells (NK) as a result of co-culture with MenSCs, relative concentrations of 41 different growth factors, their receptors, and binding proteins in the MenSCs secretome were tested using membrane-based antibody array. We have demonstrated that MenSCs secrete a large quantity of growth factors including EGF, fibroblast growth factor (FGF), platelet-derived growth factor (PDGF), TGF-β, VEGF, HGF, insulin-like growth factor (IGF)-1, and angiopoietin-1, which are essential components for immunoregulation and tissue repair by MenSCs [47]. Also, in an unpublished study conducted by this team, a comparison between the secretome content of MenSCs and BMSCs by Western blotting showed that MenSCs produced comparable amounts of HGF, VEGF, Stromal cell-derived factor (SDF)-1, Hypoxia-inducible factor (HIF)-1α, IL-1β, Angiopoietin (ANG)-1, and ANG-2 compared to the BMSCs. In this way, significantly higher levels of VEGF and HIF-1α and lower levels of IL-1β were found in the MenSCs secretome. Moreover, the MenSCs derived secretome contains lots of small EVs consisting of regulatory proteins, RNAs, and DNAs, lipids, and signaling peptides promoting regenerative repair of different tissues in various diseases including inflammatory diseases [48]. The secretome of endometrial-derived mesenchymal stromal cells contains about 900 proteins which 617 proteins are involved in activating various complement components, regulation of adaptive and innate immune reactions, antigen presentation, negative control of apoptosis, and different signaling pathway [49]. It consists certain functional immunomodulatory proteins such as colony-stimulating factor-1, PYCARD (PYD and CARD domain), and endoplasmic reticulum aminopeptidase 1 (ERAP1) that modulate immune reactions in interferon (IFN)-γ primed MenSC-derived small EVs [45]. Based on the studies, following the MenSCs license with pro-inflammatory cytokines such as IFN-γ modulations, the cargo of EV proteins was changed and the antigens processing and the presenting proteins and miRNA were increased. High levels of ICAM-1, angiogenin, angiopoetin-2, osteoprotegerin, and IL-8 have also been found in the EVs of MenSCs.

In a recent study, safety and efficacy of administration of exosomes from allogeneic BMSCs (ExoFloTM) in a small and non-randomized cohort study of patients with severe COVID-19 have been examined. This MSC-based cell free product demonstrated a good safety profile with no adverse events and was able to restore oxygenation, downregulate cytokine storm, and improve the biomarkers associated with inflammation [50]. We have shown that BMSCs and MenSCs secretome contained comparable amounts of growth factors and soluble growth factor receptors except for β-Nerve Growth Factor (NGF), VEGF-A, Insulin-like growth factor binding proteins (IGFBP)-1, IGFBP-2, IGFBP-3, and IGFBP-4 [46]. Due to some common secretory factors, close response of these two stem cells type (BMSCs and MenSCs) in therapeutic strategies is expected, while some differences observed in improvement levels of patients could be rationalized by the discrepancy in levels of mentioned growth factors in the secretome of these stem cell sources. Nevertheless, differences in age and condition of stem cells donors, dose and protocol of secretome preparation and administration procedure and route should not be ignored.

## Conclusion

This is the first known clinical study to date using MenSCs-derived secretome as treatment of patients with severe COVID-19 pneumonia. Despite the significant advantages of stem cell secretome derivatives compared to stem cell, including greater ease of production and storage, and longer shelf life of the product, the lack of regulatory requirements related to manufacturing and quality control, along with the lack of familiarity of many physicians with this type of products has caused, despite having many capabilities to use them in many limited cases.

This study showed that the injection of MenSCs secretome was safe and well-tolerated by severely ill patients. Evaluation of the effectiveness of this treatment depicted that injecting five consecutive doses of MenSCs secretome, improved hypoxia, and pulmonary involvement, restored immune system function, and controlled cytokine storm in critically ill patients and hospitalized in the ICU. Given the mechanism of action of this type of treatment, MSC-derived secretome would be a feasible therapeutic strategy for the inflammatory state; however, it would be better if therapy was performed before this phase in order to prevent or alleviate multiorgan failure. Finally, we hope that the results of this study provide a new perspective on the use of menstrual blood stromal cell secretions in the treatment of many inflammatory processes such as ARDS, chronic obstructive pulmonary disease, sepsis, autoimmune diseases, and cancer [51–56]. Further clinical trials are necessary to examine safety and efficacy.

## Data Availability

All of the data generated and analyzed during this study are included in our manuscript.

## Abbreviations

MSCs: Mesenchymal stromal cells
MenSCs: Menstrual blood Stromal Cells
SARS-CoV-2: Severe acute respiratory syndrome coronavirus 2
ARDS: Acute respiratory distress syndrome
IL: Interleukin
TNF-α: Tumor necrosis factor-α
TGF: Tumor growth factor
HGF: Hepatocyte growth factor
VEGF: Vascular endothelial growth factor
LIF: Leukemia inhibitory factor
EGF: Epidermal growth factor
NGF: Nerve growth factor
PG: Prostaglandin
IL-1Ra: Interleukin-1 receptor antagonist
mpCCL2: Metalloproteinase-Processed-C motif chemokine ligand2
BDNF: Brain-derived neurotrophic factor
GvHD: Graft versus host disease
EV: Extracellular vesicle
BM: Bone marrow
FGF: Fibroblast growth factor
PDGF: Platelet-derived growth factor
SDF: Stromal cell-derived factor
HIF: Hypoxia-inducible factor
ANG: Angiopoietin
ERAP: Endoplasmic reticulum aminopeptidase
IFN: Interferon
ICAM: Intercellular adhesion molecule
NK: Natural killer
IGFBP: Insulin-like growth factor binding proteins
cGMP: Current good manufacturing practices
CRP: C-reactive protein
LDH: Lactate dehydrogenase
CD: Cluster of differentiation
ICU: Intensive care unit
GCP: Good clinical practice
MCB: Master cell bank
FDA: Food and drug administration
PCR: Polymerase chain reaction
DVT: Deep vein thrombosis
DMEM-F12: Dulbecco's modified eagle medium/nutrient mixture F-12
PL: Platelet lysate
PBS: Phosphate-buffered saline
CBC: Complete blood count
PT/INR: Prothrombin time and international normalized ratio
LFT: Liver function tests
ESR: Erythrocyte sedimentation rate
CRP: C-reactive protein
CT: Computed tomography
GGO: Ground-glass opacification/opacity
